# HMMR/RHAMM recruits SACK1D/FAM83D-CK1α complex at the mitotic spindle to control spindle alignment

**DOI:** 10.1016/j.isci.2025.114417

**Published:** 2025-12-12

**Authors:** Tyrell N. Cartwright, Naveen K. Nakarakanti, Karen Dunbar, Luke J. Fulcher, Selina Bader, Nicola T. Wood, Thomas J. Macartney, Gopal P. Sapkota

**Affiliations:** 1 Medical Research Council Protein Phosphorylation and Ubiquitylation Unit, School of Life Sciences, University of Dundee, Dundee DD1 5EH, UK

**Keywords:** Cell biology, Molecular biology

## Abstract

The SACK1D/FAM83D-CK1α complex assembles at the mitotic spindle to orchestrate proper spindle positioning and error-free progression through mitosis. The full molecular picture of how this complex assembles and disassembles over the cell division cycle remains to be fully defined. Here, we show that hyaluronan-mediated motility receptor (HMMR) is critical for SACK1D-CK1α complex formation at the spindle, co-localizes with the SACK1D-CK1α complex throughout mitosis, and is necessary for correct mitotic spindle alignment. We find that HMMR binds to the C-terminal α-helix of SACK1D, and this helix is also important for the mitotic interaction between SACK1D and CK1α. We demonstrate that HMMR binding stabilizes SACK1D. We map the mitotic hyperphosphorylation sites on SACK1D and show that this hyperphosphorylation signals the destruction of SACK1D upon mitotic exit. The destruction also requires the C-terminal α-helix of SACK1D, suggesting that hyperphosphorylation of SACK1D in mitosis potentially exposes the C-terminal degron sequences resident on SACK1D. This study provides key molecular insights into the assembly and fate of the HMMR-SACK1D-CK1α complex at the mitotic spindle.

## Highlights

• HMMR is required for CK1α mitotic spindle recruitment
• The c-terminal α-helix of SACK1D is responsible for its association with HMMR
• HMMR stabilizes SACK1D by binding its c-terminal α-helix
• The c terminus and phosphorylation coordinate SACK1D post mitotic destruction

## Graphical abstract

**Figure:**
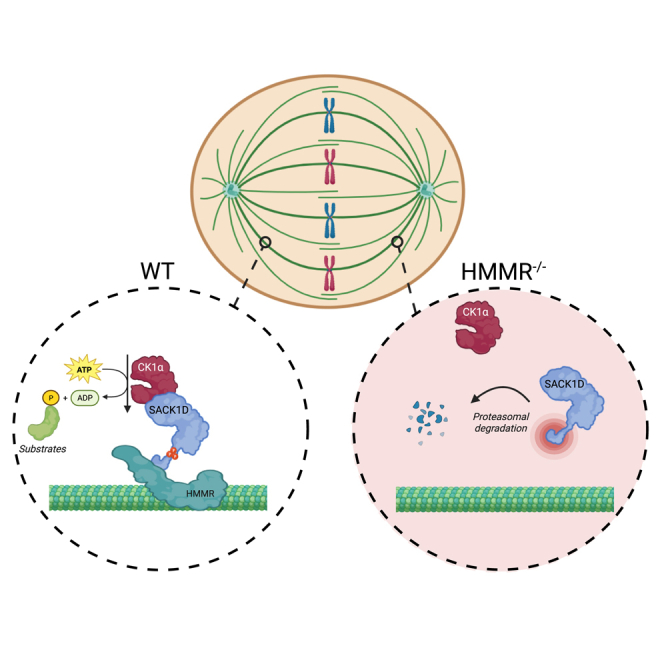

## Introduction

Scaffold Anchor of CK1 (SACK1) domain-containing protein D (SACK1D; known previously as FAM83D or CHICA) is a microtubule-localized scaffold protein conserved in vertebrates that orchestrates proper spindle alignment, ensuring timely cell division.^1^ In mitosis, SACK1D directly associates with and recruits the Ser/Thr protein kinase CK1α to the mitotic spindle via its conserved SACK1 domain (previously known as domain of unknown function 1669, DUF1669).^1^^,^^2^ We previously showed that CK1α kinase activity at the mitotic spindle is essential for correct spindle positioning and timely, error-free progression through mitosis, and that CK1α recruitment to the mitotic spindle is dependent on SACK1D.^1^ More recently, and broadly, CK1 isoforms have been reported to regulate microtubule dynamics, for example though phosphorylation of Fam110A, chromosomal alignment and spindle formation.^3^^,^^4^^,^^5^ We and others have also shown that SACK1D is recruited to the mitotic spindle through its association with the microtubule-associated protein hyaluronan-mediated motility receptor (HMMR, aka RHAMM or CD168).^1^^,^^6^^,^^7^ However, the precise molecular mechanisms by which HMMR binds to and recruits SACK1D to the mitotic spindle remain to be defined.

HMMR, first identified as a receptor for hyaluronic acid, has been implicated in a wide range of cancers with high expression correlated to poor outcomes. Several studies on hepatocellular carcinoma,^8^^,^^9^ breast cancer,^10^ lung adenocarcinoma,^11^ acute lymphoblastic leukemia,^12^ and head and neck squamous cell carcinoma^13^ point to HMMR playing an important role in cancer cell proliferation. While some studies have linked this to hyaluronan signaling pathways, most studies have focused on HMMR as a prognostic indicator and have not examined the underpinning molecular pathways that HMMR controls. Studies examining the molecular roles of HMMR have shown that it acts intracellularly in concert with other microtubule and centrosomal proteins to govern structural cytoskeletal, mitotic, and meiotic processes, which directly affect cell proliferation, correct cell division cycle progression and microtubule organization.^14^ Indeed, HMMR was shown to regulate microtubule nucleation and spindle assembly in *Xenopus*.^15^ HMMR has been shown to play an important role in the Polo-like kinase (PLK)-dependent spindle positioning pathway and has been shown to restrict the activity of the kinesin Eg5 by modulating TPX2 recruitment.^7^^,^^16^ HMMR also plays a role outside of mitosis in regulating TPX2 localization in neurons.^17^ Some studies have highlighted the importance of HMMR in other aspects of proliferation and mitosis.^18^^,^^19^ Structurally, HMMR contains a leucine zipper motif at its C terminus, which was shown to be important for centrosomal localization.^20^ The N terminus of HMMR, on the other hand, is known to be important for microtubule binding, as deletion of the first 189 residues of the protein abolished its localization to microtubules.^6^ Though these studies paint a clear picture of HMMR as an intracellular, cytoskeletal, and mitotic factor, the regulation bestowed by HMMR to the molecular mechanism underpinning CK1α recruitment to the mitotic spindle remains undefined.

SACK1D and HMMR are both cell cycle regulated proteins. Both proteins are upregulated in G2/M and are degraded upon mitotic exit, temporally coincident with other critical mitotic proteins, although precisely how this is achieved is not known.^1^ Through the interaction with its substrate binder Cdc20, the anaphase-promoting complex/cyclosome (APC/C) E3 ligase complex is responsible for the initiation of anaphase onset through the destruction of key substrates including both Cyclin B1, the activation cofactor of cyclin-dependent kinase 1 (CDK1), which is responsible for a myriad of critical mitotic phosphorylation events, and securin, an inhibitor of separase, which is responsible for cohesin cleavage allowing sister chromatids to separate.^21^ Recruitment to the APC/C requires the recognition of one of several destruction sequence motifs on target proteins, such as the D-box (RxxLxxxxN), KEN, and ABBA motifs.^21^ Given the precise temporal control of both SACK1D and HMMR expression, it is possible that these too are targets of the APC/C, and as such contain mitotic degrons resembling those known to facilitate destruction at anaphase onset. Additionally, SACK1D undergoes hyperphosphorylation in mitosis leading to an apparent molecular weight shift by SDS-PAGE of ∼25 KDa, and this requires CK1α kinase activity.^1^ However, the precise sites of mitotic hyperphosphorylation on SACK1D and what effect hyperphosphorylation has on SACK1D function or fate remain undefined. In this study, we investigate the molecular details underpinning precisely how HMMR recruits SACK1D-CK1α complex to the mitotic spindle. Finally, we examine mitotic hyperphosphorylation of SACK1D and its role in the post-mitotic degradation of SACK1D and HMMR.

## Results

### HMMR co-localizes with SACK1D-CK1α complex at the spindle throughout mitosis and is required for SACK1D-CK1α complex formation and correct mitotic spindle alignment

**Figure 1:**
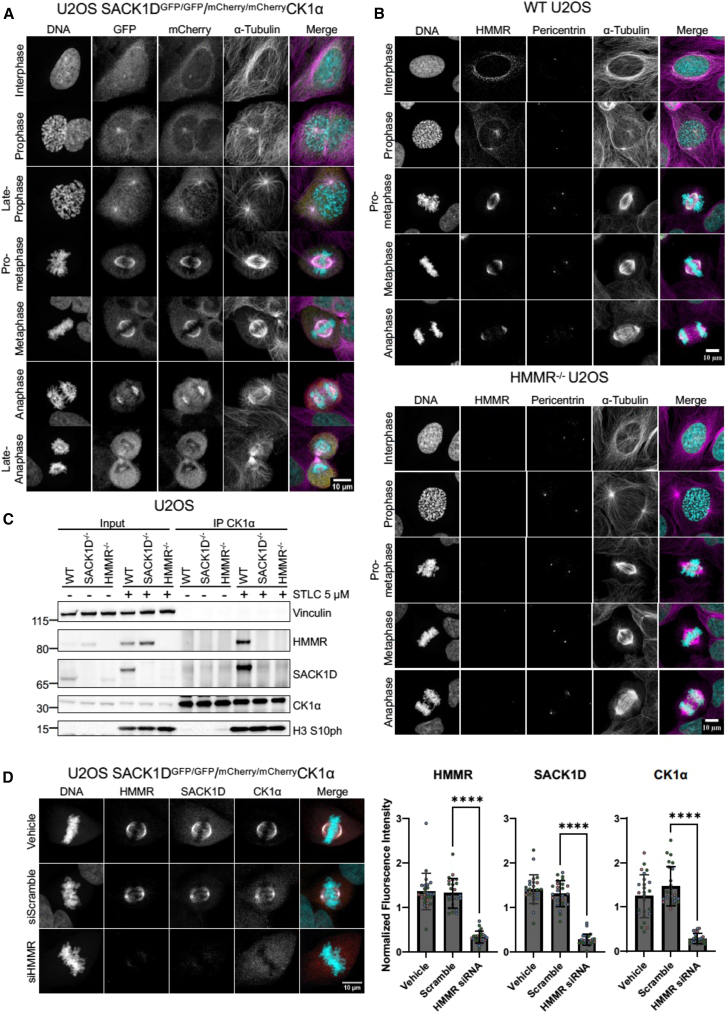
HMMR is a critical component of the SACK1D:CK1α spindle complex (A) Representative confocal IF microscopy images of SACK1D^*GFP/GFP*^ and CK1α^*mCherry/mCherry*^ double knock-in U2OS cells showing that SACK1D-GFP and mCherry-CK1α localize to the centrosomes and mitotic spindle throughout various stages of mitosis. (Scale bars, 10 μm). (B) Representative confocal IF microscopy images of WT and HMMR^−/−^ U2OS cells showing that HMMR localizes to the centrosomes and mitotic spindle throughout various stages of mitosis. (Scale bars, 10 μm). (C) Representative western blot with the indicated antibodies of lysate inputs and CK1α immunoprecipitates (IPs) in WT, SACK1D^−/−^, and HMMR^−/−^ U2OS cells under asynchronous conditions or those treated with STLC at 5 μM for 16 h to induce mitotic arrest. H3S10ph observed in mitotic anti-CK1α IPs is potentially due to beads being subject to low stringency washes and/or the high sensitivity of the antibody detecting low levels of contaminating mitotic H3S10ph in the beads but it is likely to be a non-specific interaction. (D) Representative confocal IF microscopy images of SACK1D^*GFP/GFP*^ and CK1α^*mCherry/mCherry*^ CRISPR double knock in U2OS cells treated with HMMR siRNA and stained with anti-HMMR antibody. Graph indicates quantification of fluorescence intensity for each protein labeled in three independent experiments (number of cells *n* = 25). Dots indicate individual cells with colors representing independent experiments, mean ± S.D., scale bars, 10 μm, *t* test ∗∗∗∗*p* < 0.001.

**Figure 2:**
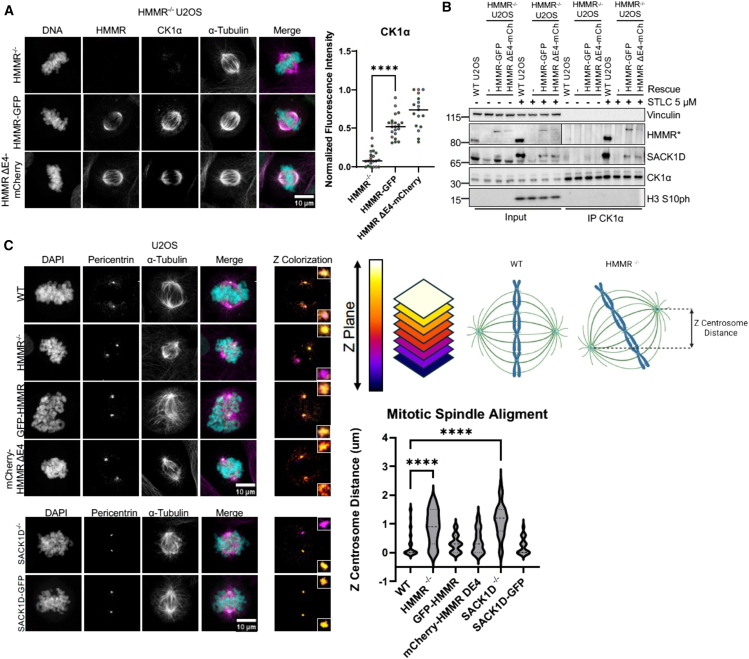
Restoration of HMMR in HMMR^−/−^ U2OS cells is sufficient to re-localize the SACK1D-CK1α complex to mitotic spindles and correct spindle alignment defects (A) Representative confocal IF microscopy images of HMMR^−/−^ U2OS cells either untransduced or transduced with retroviruses to stably express HMMR-GFP or HMMR-GFP lacking exon 4 (ΔE4-GFP) and stained for HMMR, CK1α and α-tubulin as indicated. Graph indicates quantification of fluorescence intensity for 3 independent experiments (number of cells *n* ≥ 17). Dots indicate individual cells with colors representing each independent biological replicate, mean ± S.D., scale bars, 10 μm, *t* test ∗∗∗∗*p* < 0.001. (B) Representative western blot analysis with the indicated antibodies of lysate inputs and CK1α IPs in WT U2OS cells and HMMR^−/−^ U2OS cells stably expressing HMMR-GFP or HMMR-GFP lacking exon 4 (ΔE4-GFP). (C) Representative images from confocal IF spindle alignment assay of WT, HMMR^−/−^, and SACK1D^−/−^ U2OS cells that were either left untransduced or transduced with retroviruses to stably express HMMR-GFP or ΔE4-GFP (in HMMR^−/−^ cells) or SACK1D-GFP (in SACK1D^−/−^ cells) as indicated. Cells were stained with Pericentrin as a marker for centrosomes. Z-colorization scheme and images show the relative positioning of each of the two centrosomes (inset zoomed for clarity) for each cell line as indicated. The Z-centrosome distance, which indicates variance between the two centrosomes across the Z-plane and is expected to be 0 if the spindles are aligned properly, were measured and plotted on a violin plot which indicates data from three independent experiments mean ± S.D., number of cells *n* ≥ 24, scale bars, 10 μm, one-way ANOVA ∗∗∗∗*p* < 0.001.

We and others have previously reported that SACK1D localizes to the mitotic spindle^1^^,^^6^ and is required for the recruitment of CK1α to the mitotic spindle to ensure correct spindle alignment and timely mitosis.^1^ We monitored the subcellular distribution of SACK1D and CK1α in unperturbed asynchronous SACK1D^*GFP/GFP*^ and CK1α^*mCherry/mCherry*^ double knock-in U2OS cells,^1^ generated through CRISPR/Cas9 genome editing, with confocal immunofluorescence (IF) microscopy through various stages of the cell cycle (Figure 1A). Both SACK1D and CK1α display diffused expression in interphase cells. At the G2/M transition, both proteins begin to coalesce around centrosomes. By late prophase/early prometaphase, when the centrosomes have separated, both proteins are strongly associated with newly forming spindle fibers protruding from the centrosomes. This association remains strong throughout spindle assembly and persists all the way until anaphase, where both proteins appear to dissociate from the spindle as chromosomes segregate (Figure 1A). Similarly, HMMR displays identical spatiotemporal localization to SACK1D and CK1α, except in non-mitotic cells, where HMMR also stains interphase microtubules (Figure 1B). Absence of IF signal for HMMR in HMMR^−/−^ U2OS cells, which were generated by CRISPR/Cas9 genome editing and validated by genomic sequencing (Figure S1), confirmed the specificity of the antibody used for IF (Figure 1B). Furthermore, when we analyzed mitotic SACK1D-CK1α complex formation by immunoprecipitating CK1α, we confirmed the absolute requirement for the presence of HMMR, as the CK1α IPs failed to pull down SACK1D in HMMR^−/−^ cells, although it should be noted that SACK1D abundance in HMMR^−/−^ cells is severely attenuated compared to WT controls (Figure 1C). Similarly, HMMR did not co-precipitate with CK1α in SACK1D^−/−^ cells (Figure 1C). In WT cells, both SACK1D and HMMR co-precipitated with CK1α specifically in mitosis (Figure 1C). We further confirmed the requirement of HMMR in mitotic SACK1D-CK1α complex formation, as siRNA-mediated depletion of HMMR in SACK1D^*GFP/GFP*^ and CK1α^*mCherry/mCherry*^ double knock in U2OS cells completely abolished the mitotic spindle accumulation of the SACK1D-CK1α complex seen in control conditions (Figure 1D). Similarly, HMMR^−/−^ U2OS cells failed to recruit SACK1D and CK1α to the mitotic spindle (Figure 2A). However, retroviral-mediated restoration of the two reported transcriptional variants of HMMR, namely the full-length HMMR-GFP and a shorter variant lacking exon 4 (HMMR ΔE4-GFP), both restored CK1α and SACK1D at the mitotic spindle (Figure 2A). Consistent with these observations, CK1α co-precipitated SACK1D and HMMR in mitotic HMMR^−/−^ U2OS cells restored with either HMMR-GFP or HMMR ΔE4-GFP, but not in control HMMR^−/−^ U2OS cells (Figure 2B). Finally, we wanted to determine the effect of HMMR loss on the spindle alignment phenotype, which we previously reported was disrupted upon the loss of SACK1D or CK1α from the spindle.^1^ We performed mitotic spindle alignment assays using confocal microscopy by performing Z-sectioning at 0.3 μm through cells to locate the centrosomes in each cell and measure their vertical separation. We found that WT U2OS cells on average showed spindles parallel to the culture surface, with little to no vertical separation. In contrast, HMMR^−/−^ U2OS cells displayed randomly rotated spindles defined by profound vertical separation during late prometaphase, suggesting a significant misalignment of mitotic spindles in HMMR^−/−^ cells. This spindle misalignment phenotype observed in HMMR^−/−^ U2OS cells was corrected by restoration of HMMR-GFP or its ΔE4 variant (Figure 2C).

### The C-terminal α-helix of SACK1D is responsible for its association with HMMR as well as regulating its mitotic-specific association with CK1α

**Figure 3:**
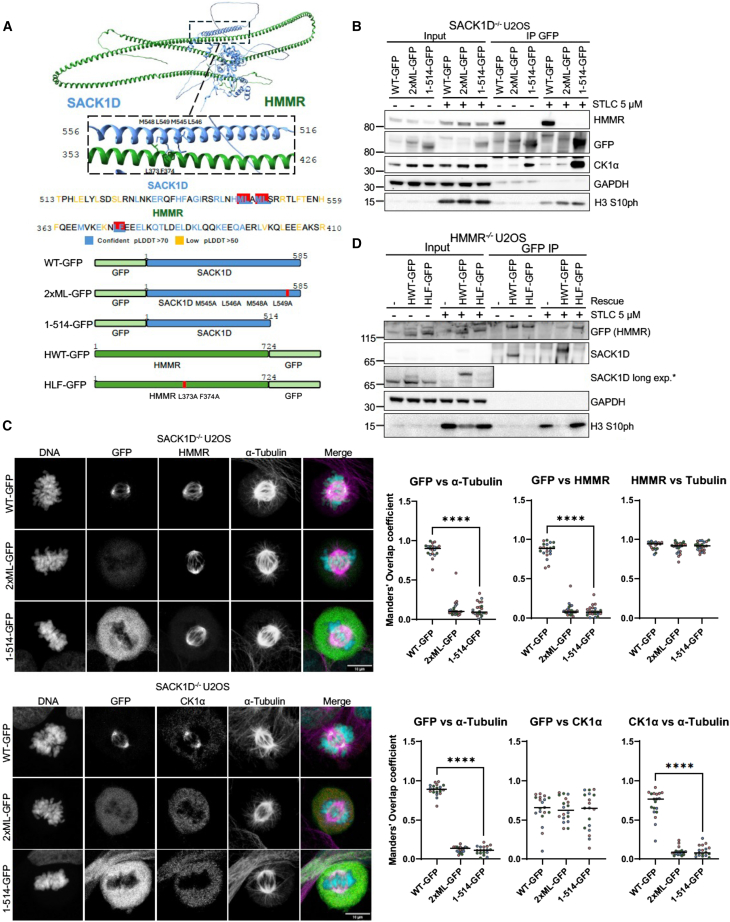
The C-terminal alpha-helix of SACK1D mediates its interaction with HMMR for spindle localization and confers mitotic specificity to its binding with CK1α (A) Representation of AlphaFold3 SACK1D-HMMR binding predictions with possible binding interface highlighted (top). Amino acids in the sequence colored based on pLDDT confidence of residue’s involvement in mediating the interaction (middle). The bottom panels show schematics of the SACK1D and HMMR constructs, designed based on AlphaFold3 structural predictions. (B) Representative western blot analysis with the indicated antibodies of lysate inputs and GFP-SACK1D IPs from SACK1D^−/−^ U2OS cells expressing the indicated SACK1D-GFP variants (2xML: SACK1D^M545A/L546A/M548A/L549A^) in asynchronous cells or those treated with STLC at 5 μM for 16 h to induce mitotic arrest. H3S10ph observed in mitotic anti-CK1α IPs is potentially due to beads being subject to low stringency washes and/or the high sensitivity of the antibody detecting low levels of contaminating mitotic H3S10ph in the beads but it is likely to be a non-specific interaction. (C) Representative confocal microscopy images of indicated GFP-SACK1D mutants expressed in SACK1D^−/−^ U2OS cells. Graphs indicate quantification of widefield IF indicating colocalization of indicated proteins using Mander Overlap Coefficient for each protein labeled in 3 independent experiments (number of cells *n* ≥ 20). Dots indicate individual cells with colors representing independent experiments, mean ± S.D., scale bars, 10 μm, *t* test ∗∗∗∗*p* < 0.001. (D) Representative western blot analysis with the indicated antibodies of lysate inputs and GFP IPs of HMMR-GFP variants (HWT: HMMR-WT; HLF: HMMR^L373A/F374A^) restored in HMMR^−/−^ U2OS cells as labeled in asynchronous cells, or those treated with STLC at 5 μM for 16 h to induce mitotic arrest. H3S10ph observed in mitotic anti-CK1α IPs is potentially due to beads being subject to low stringency washes and/or the high sensitivity of the antibody detecting low levels of contaminating mitotic H3S10ph in the beads but it is likely to be a non-specific interaction.

To better understand how HMMR controls the role of the SACK1D-CK1α complex in ensuring proper spindle alignment, we set out to determine how SACK1D interacts with HMMR. Using AlphaFold3,^22^ the interaction between HMMR and SACK1D was predicted to involve the C-terminal α-helix of SACK1D and residues within the central coiled-coil region of HMMR (Figures S2A and S2B). Guided by the predicted structure, a series of mutations within the region of SACK1D predicted to disrupt HMMR binding or a truncated fragment (aa1-514) lacking the entire C-terminal α-helix were generated and their ability to interact with HMMR investigated (Figure 3A). SACK1D-GFP (WT-GFP), SACK1D^*M545A,L546A,M548A,L549A*^-GFP (2xML-GFP), and SACK1D^*1-514*^-GFP (1-514-GFP) were expressed in SACK1D^−/−^ U2OS cells and immunoprecipitated using an anti-GFP nanobody immobilized on Sepharose beads. WT SACK1D-GFP was able to co-precipitate HMMR in both interphase and mitotic cells. However, neither 2xML-GFP nor 1-514-GFP were able to co-precipitate HMMR (Figure 3B), suggesting that the C-terminal α-helix of SACK1D is essential for mediating the interaction with HMMR. Another noteworthy observation is that the 2xML-GFP mutant, while abundant in interphase, displayed severely depleted levels in mitosis (Figure 3B). Surprisingly, unlike wild-type SACK1D which binds CK1α only in mitosis, both 2xML-GFP and 1-514-GFP mutants bound CK1α regardless of cell cycle stage (Figure 3B). By fluorescence microscopy, a loss of spindle localized SACK1D and CK1α was observed for cells expressing either the 2xML-GFP and 1-514-GFP SACK1D mutants. In contrast, CK1α and HMMR co-localized to the mitotic spindle in cells expressing the wild-type SACK1D-GFP control (Figure 3C). Reciprocally, guided by the Alpahfold3 prediction of the HMMR-SACK1D interaction interface (Figure S2), we generated a HMMR^*L373A,F374A*^-GFP (HLF-GFP) mutant to disrupt the predicted interaction with SACK1D (Figure 3A). Indeed, while WT HMMR-GFP (HWT-GFP) restored in HMMR^−/−^ U2OS cells binds SACK1D, the HLF-GFP mutant abolished SACK1D binding in both asynchronous and mitotic cells (Figure 3D). Interestingly, we also noted that restoration of HMMR (HWT-GFP) in HMMR^−/−^ U2OS cells rescued basal SACK1D levels that were reduced in HMMR^−/−^ cells in asynchronous conditions and fully restored the mitotic electrophoretic mobility shift of SACK1D, indicative of its mitotic hyperphosphorylation (Figure 3D). However, the HLF-GFP mutant was unable to rescue basal SACK1D levels, or its hyperphosphorylation in mitosis (Figure 3D). This is consistent with the observed rescue of SACK1D levels and mitotic phosphorylation upon restoration of HMMR-GFP and HMMRΔE4-GFP in HMMR^−/−^ cells (Figure 2B).

### HMMR stabilizes full length SACK1D

**Figure 4:**
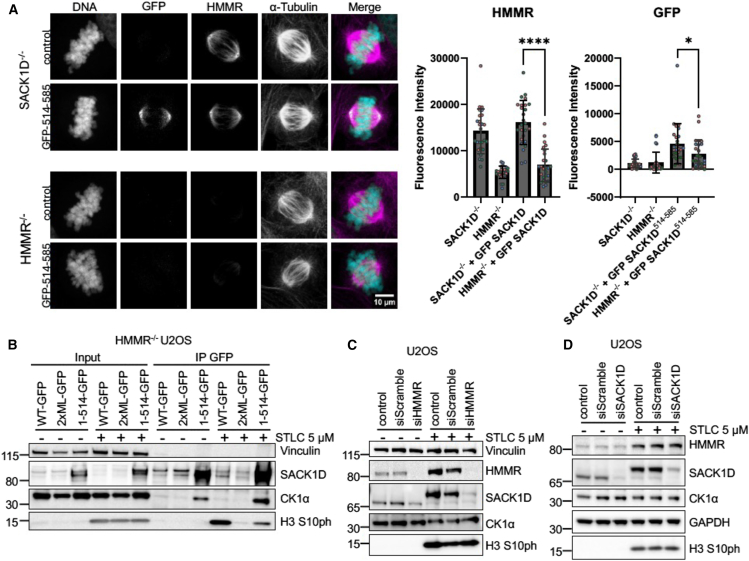
HMMR is essential for the stabilization of SACK1D, especially in mitosis (A) Representative confocal IF microscopy images of SACK1D^−/−^ and HMMR^−/−^ U2OS cells expressing GFP-FAM83^514−585^. Graph indicates relative florescence intensity of indicated protein on α-tubulin representative of three independent experiments. Dots indicate individual cells with different colors representing independent experiments, mean ± S.D., number of cells *n* = 26, scale bars, 10 μm, *t* test ∗∗∗∗*p* < 0.001. (B) Representative western blot analysis with the indicated antibodies of lysate inputs and GFP IPs from HMMR^−/−^ U2OS cells expressing the indicated SACK1D-GFP variants in asynchronous or those treated with STLC at 5 μM for 16 h to induce mitotic arrest. (C) Representative western blot analysis with the indicated antibodies of lysate inputs from WT U2OS cells transfected with HMMR siRNA for 24 h and lysed in asynchronous or mitotic (5 μM STLC for 16 h) conditions. (D) Representative western blot analysis with the indicated antibodies of lysate inputs from WT U2OS cells transfected with SACK1D siRNAs for 24 h and lysed in asynchronous or mitotic (5 μM STLC for 16 h) conditions.

To provide further evidence for the role of HMMR in recruiting SACK1D to the spindle via direct interaction with SACK1D, the HMMR binding fragment of SACK1D (aa514-585) was fused to GFP and expressed in SACK1D^−/−^ and HMMR^−/−^ U20S cells. As shown by representative confocal microscopy images, GFP-SACK1D^514-585^ restored in SACK1D^−/−^ cells is recruited to the mitotic spindle but not in HMMR^−/−^ cells (Figure 4A). As this provides further evidence for this region on SACK1D being responsible for HMMR interaction, to understand the extent of the role HMMR plays in SACK1D-CK1α complex formation and apparent SACK1D stabilization, WT-GFP, 2xML-GFP, and 1-514-GFP were also expressed in HMMR^−/−^ U2OS cells (Figure 4B). As expected, we saw no CK1α co-precipitation with WT-GFP IPs in the absence of HMMR, neither in interphase nor mitotic cells (Figure 4B). Surprisingly, 2xML-GFP IPs did not co-precipitate CK1α but 1-514-GFP SACK1D IPs co-precipitated CK1α, independent of cell cycle stage (Figure 4B). On closer inspection, we noticed a substantially lower abundance of both WT-GFP and 2xML-GFP in HMMR^−/−^ U2OS cells compared to 1-514-GFP (Figure 4B). This reduction in abundance appeared to be further exacerbated in mitosis and suggests that HMMR stabilizes full-length SACK1D through direct interaction (Figure 4B). Consistent with this notion, we note that HMMR^−/−^ cells display lower levels of SACK1D protein compared to WT U2OS cells under asynchronous conditions, while in mitosis this difference in SACK1D abundance is further exacerbated as almost no SACK1D can be detected in mitotic HMMR^−/−^ cells (Figure 1C). This may also explain our previous observations of why 2xML-GFP mutants are destabilized in mitosis (Figure 3B) and the low abundance of WT SACK1D-GFP and 2xML-GFP in HMMR^−/−^ cells along with their apparent further mitotic destabilization (Figure 4B). The evidence for HMMR regulating SACK1D stability, while compelling, is indirect and relies mostly on a single HMMR^−/−^ CRISPR clone, which may have irreversibly altered protein expression and/or signaling. To test the theory that HMMR regulates SACK1D stability directly, HMMR was depleted using siRNAs in WT U2OS cells and SACK1D abundance examined (Figure 4C). Indeed, the loss of HMMR leads to a robust reduction in SACK1D levels, with a further decrease in levels observed in mitotic cells (Figure 4C). Paradoxically, ablation of SACK1D appeared to lead to an increased accumulation of HMMR (Figure 1D). However, this was not reproducible when siRNAs were used to deplete SACK1D (Figure 4D), suggesting SACK1D is unlikely to regulate the stability of HMMR in the same way that HMMR regulates SACK1D stability. Furthermore, loss of SACK1D did not affect the rate of HMMR turnover upon mitotic exit (Figure S2C). In WT U2OS cells, we see a robust mitotic SACK1D phosphorylation and after 3–5 h of STLC washout, we observe almost complete and synchronous degradation of both SACK1D and HMMR (Figure S2C). In SACK1D^−/−^ cells, although there is an increase in basal and mitotic HMMR levels (a likely CRISPR artifact), the rate and extent of post-mitotic HMMR degradation did not alter substantially compared to WT cells (Figure S2C).

### Mitotic phosphorylation of SACK1D and its C-terminal α-helix signal its destruction upon mitotic exit

**Figure 5:**
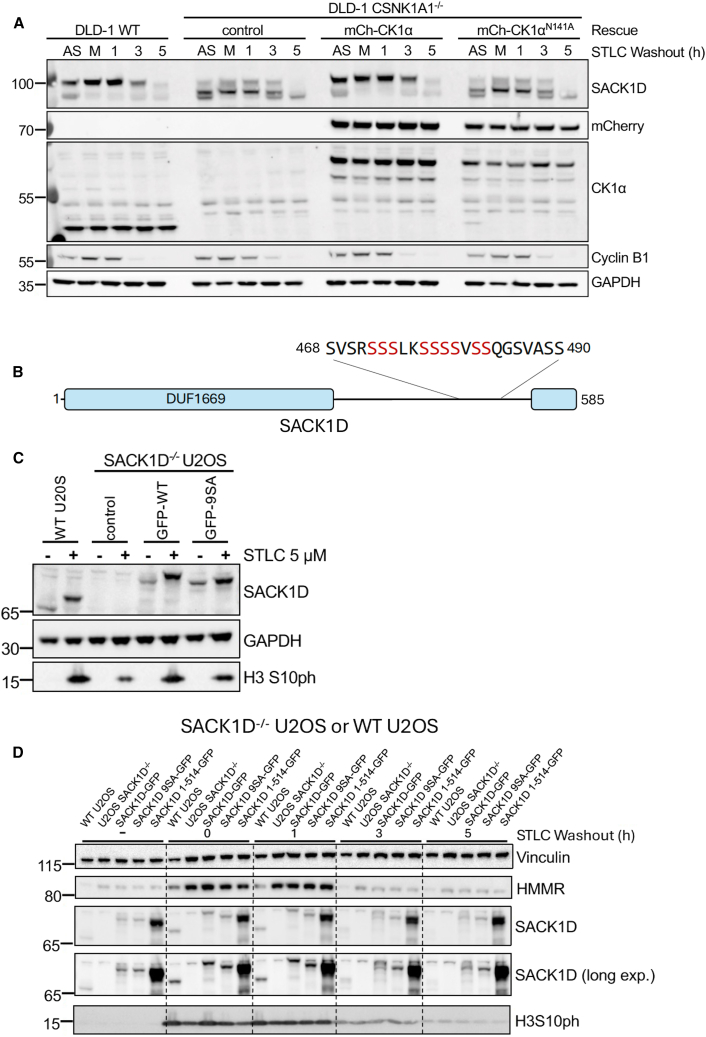
CK1α-dependant phosphorylation of SACK1D and its C-terminal α-helix mark SACK1D for rapid post-mitotic destruction (A) Representative western blot analysis with the indicated antibodies of lysate inputs from an STLC washout assay as indicated. WT DLD-1, *CSNK1A1*^−/−^ DLD-1, or *CSNK1A1*^−/−^ DLD-1 cells stably expressing either mCherry-CK1α or catalytically inactive mCherry-CK1α^N141A^ mutant were left untreated (AS) or treated with STLC at 5 μM for 16 h to induce mitotic arrest, mitotic cells isolated by shake-off and plated in fresh medium without STLC prior to lysis at the indicated time post mitotic shake-off. Cyclin B1 was employed as a control for post-mitotic degradation. (B) Schematic of SACK1D showing the location of its serine rich region which determine the mitotic hyperphosphorylation-induced electrophoretic mobility shift by western blotting. (C) Representative western blot analysis with the indicated antibodies of lysate inputs from WT U2OS and SACK1D^−/−^ U2OS cells expressing GFP-SACK1D or GFP-SACK1D^9SA^ (9 Ser residues indicated in B in red mutated to Ala) in asynchronous and mitotic cells (5 μM STLC for 16 h). (D) Representative western blot analysis with the indicated antibodies of lysate inputs from an STLC washout assay (as in A) using WT U2OS cells and SACK1D^−/−^ U2OS cells or those expressing the indicated GFP-SACK1D constructs in asynchronous and mitotic cells (5 μM STLC for 16 h).

As shown above, and consistent with previous observations,^1^ SACK1D is hyperphosphorylated in mitosis. Although we found that the interaction between SACK1D and CK1α was essential to drive this mitotic SACK1D hyperphosphorylation, the precise role of CK1α in SACK1D phosphorylation and the function of these phosphorylation events remains to be determined. To more closely examine the role of CK1α and its catalytic activity on SACK1D hyperphosphorylation, we generated *CSNK1A1*^−/−^ DLD-1 colorectal cells by CRISPR/Cas9 genome editing,^23^ as repeated efforts to generate *CSNK1A1*^−/−^ U2OS cells with CRISPR/Cas9 genome editing failed, potentially suggesting genetic ablation of CK1α is lethal in this background. We employed *CSNK1A1*^−/−^ DLD-1 cells and stably restored the expression of either WT mCherry(mCh)-CK1α or a catalytically inactive mCh-CK1α^*N141A*^ mutant (Figure 5A). Unlike in WT U2OS cells where SACK1D runs at ∼75 kDa in asynchronous extracts and ∼100 kDa in mitotic cells (Figure 1C), SACK1D was evident at both ∼75 kDa and ∼100 kDa in asynchronous WT DLD-1 cells, potentially suggesting a high mitotic index in asynchronous conditions (Figure 5A). Despite this, in mitotic WT DLD-1 cells, only the hyperphosphorylated SACK1D migrating at ∼100 kDa was evident (Figure 5A). This mobility shift of SACK1D in mitosis in WT DLD-1 cells was severely impaired in *CSNK1A1*^−/−^ DLD-1 cells (Figure 5A). Restoration of WT mCh-CK1α in *CSNK1A1*^−/−^ cells but not the catalytically inactive mCh-CK1α^*N141A*^ mutant completely rescued the mitotic hyperphosphorylation of SACK1D comparable to that seen in WT DLD-1 cells (Figure 5A), suggesting that the catalytic activity of CK1α is essential for mitotic SACK1D hyperphosphorylation. Furthermore, we also noticed that post-mitotic destruction of SACK1D observed in WT and *CSNK1A1*^−/−^ DLD-1 cells rescued with WT mCh-CK1α was impaired in *CSNK1A1*^−/−^ DLD-1 cells or those rescued with the catalytically inactive mCh-CK1α^*N141A*^ mutant (Figure 5A). These observations prompted us to hypothesize that SACK1D hyperphosphorylation by CK1α in mitosis may promote its destruction. To investigate this, we employed a phospho-proteomic analysis on mitotic SACK1D followed by site-directed mutagenesis of enriched phospho-sites (Figures S3A–S3C). This allowed us to rule out the involvement of 4 identified pS/pT residues in the observed mitotic SACK1D mobility shift (Figures S3A–S3C). Considering that the hyperphosphorylated mitotic phospho-peptide(s) responsible for the profound mitotic mobility shift may have evaded detection by mass spectrometry, potentially due to poor ionization, we focused on a Ser-rich region of SACK1D (Figure S3D). We then systematically mutated 9 serine residues to alanine, either individually or collectively (Figure S3E). When all 9 serine residues were mutated to alanine (SACK1D-9SA), we observed a near-complete disappearance of the mitotic SACK1D hyperphosphorylation (Figure 5C), although none of the individual point mutations altered the mitotic mobility shift (Figure S3E). Interestingly, the post-mitotic destruction of SACK1D-9SA was slightly less efficient than WT SACK1D (Figure 5D), suggesting that CK1α-dependent phosphorylation of SACK1D in mitosis potentially triggers its degradation upon mitotic exit. Given the reduced SACK1D abundance observed in HMMR^−/−^ U2OS cells and the concomitant high abundance of the SACK1D^1-514^ fragment when expressed in SACK1D^−/−^ cells, we postulated that the C terminus of SACK1D, in addition to mediating HMMR binding, may also be crucial for its stability. Indeed, compared to WT SACK1D-GFP, which showed robust post-mitotic degradation, SACK1D^1-514^-GFP restored in SACK1D^−/−^ cells displayed an impaired post-mitotic degradation, despite its ability to bind to CK1α independently of the mitotic context (Figure 5E). These data suggest that the phosphorylation of the serine-rich region of the full-length SACK1D potentially triggers the recognition of the mitotic degron sequences situated at the C terminus of SACK1D in the same region where it binds HMMR.

## Discussion

**Figure 6:**
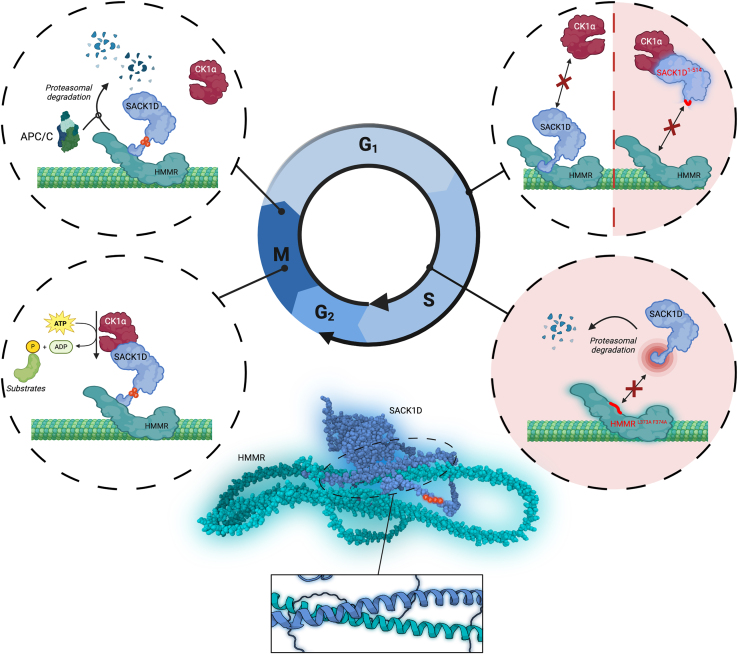
A graphical summary of key findings and the molecular insights elucidated by this study Created under license using Biorender (www.biorender.com).

In this study, we shed light on the intricate molecular interplay involved in the correct spatiotemporal recruitment of CK1α to the mitotic spindle in dividing cells (Figure 6). We demonstrate that the microtubule-associated protein HMMR is responsible for recruiting SACK1D to microtubules and establish that a coiled-coil region comprising residues 363–410 of HMMR (specifically L373 & F374) interacts with an α-helix of SACK1D comprising residues 516–556 (specifically M545-L549). Disrupting several residues within these regions completely abolishes HMMR-SACK1D binding as well as the spindle localization of SACK1D. This is consistent with a previous study which showed that deleting a large fragment from HMMR comprising aa365-546 abolished SACK1D binding and similarly, deleting aa383-585 from SACK1D impeded interaction with HMMR.^6^ SACK1D recruits CK1α to the mitotic spindle.^1^ Ablation of HMMR from cells or restoration of SACK1D mutants that no longer bind to HMMR in SACK1D KO cells completely block the localization of CK1α to the mitotic spindle, suggesting that HMMR is crucial for the recruitment of the SACK1D-CK1α complex to the mitotic spindle. Previously, we had shown that when the spindle localization of CK1α was blocked, either via SACK1D ablation or via knocking in a SACK1D^F283A^ mutation that is incapable of binding CK1α, the cells displayed impaired spindle alignment and delayed mitotic progression.^1^ Consistent with this, here we find that ablation of HMMR, which leads to the absence of SACK1D-CK1α complex at the mitotic spindle, also causes misalignment of the mitotic spindles. Our data suggests that the principal role of HMMR in mitosis is to recruit the SACK1D-CK1α complex to the mitotic spindle to control proper spindle positioning.

Peculiarly, disrupting the SACK1D-HMMR interaction by removing or mutating the C-terminal coiled-coil region of SACK1D led to its constitutive binding to CK1α, independent of the mitotic context. This further strengthens the argument that the C terminus of SACK1D acts as its own negative regulator of CK1α binding in interphase and implies that its interaction with HMMR is integral to this negative regulatory mechanism.^1^ Removal of the C-terminal helix also led to stabilization of SACK1D, suggesting the presence of degron sequences at the C terminus of SACK1D. Consistent with this notion, mutating the C-terminal helix residues (2xML->AA) from SACK1D to abolish the HMMR interaction while retaining the rest of the C terminus, resulted in reduced stability compared to wild-type SACK1D. This implies that the C terminus of SACK1D not only contains the degron motifs but that they are protected when SACK1D is bound to HMMR. HMMR appears to be critical for the stabilization of full-length SACK1D, especially in mitosis. This means that in any context where cells lack HMMR or harbor a mutant HMMR that cannot interact with SACK1D, the spindle defects that are associated with the absence of SACK1D-CK1α complex at the spindle would also persist due to the severe attenuation of SACK1D protein levels. However, ablation of SACK1D, which results in the absence of CK1α from the spindle, does not appear to impact HMMR stability or its post-mitotic destruction, suggesting that the SACK1D-CK1α complex does not directly regulate HMMR stability in mitosis.

We show that mitotic SACK1D hyperphosphorylation plays a role in its stability. Loss of SACK1D phosphorylation by the genetic ablation of CK1α (the kinase responsible for most of the electrophoretic mobility shift on SACK1D observed in mitosis) or mutating 9 serine residues responsible for the mitotic SACK1D mobility shift protect SACK1D from post-mitotic destruction. This is consistent with our previous observations whereby targeted dephosphorylation of SACK1D via recruitment of PPP1CA and PPP2CA, which caused a collapse of the mitotic SACK1D hyperphosphorylation electrophoretic mobility shift, impeded the rate of post-mitotic destruction of SACK1D.^24^ Unlike SACK1D and HMMR, CK1α itself is not degraded over the course of cell division cycle. Together, these data suggest a highly regulated mechanism by which SACK1D recruits CK1α to the mitotic spindle and ensures CK1α is not mislocalized following mitosis. Any SACK1D not bound to HMMR is degraded to potentially avoid sequestration of CK1α and any SACK1D which has been activated and binds CK1α is subsequently heavily phosphorylated and primed for degradation so that CK1α can be released back into the cytoplasmic pool and carry out other functions. In mitosis, it is through both the hyperphosphorylation of SACK1D and destruction of HMMR that SACK1D may become exposed for its destruction upon mitotic exit. Closer inspection of the C-terminal region on SACK1D reveals multiple putative D-box motifs that could potentially engage with the APC/C via Cdc20.^25^ Expression of the C terminus of SACK1D containing putative D-box motifs fused to GFP leads to stable expression and spindle accumulation of GFP only in the presence of HMMR, whereas very low expression is observed in HMMR^−/−^ cells. Similarly, Alphafold3 predicts the interaction of HMMR with Cdc20 via a KEN motif providing a mechanism for destruction of HMMR by the APC/C.

CK1α is reported to control a plethora of cellular processes, including WNT, Hippo, and Sonic Hedgehog (Shh) signaling, cell survival, and apoptosis and, as shown here, the cell division cycle.^26^ For one protein to accomplish these diverse roles, intricate regulation of the spatiotemporal localization of CK1α is necessary and in mammalian cells, the eight members of the SACK1 domain-containing proteins play a critical role in this context.^1^^,^^2^^,^^26^^,^^27^^,^^28^^,^^29^^,^^30^^,^^31^^,^^32^^,^^33^ Among the SACK1(A-H) proteins, SACK1D is unique in that its interaction with CK1α requires the mitotic context and is inhibited during interphase. This implies that the mitotic context, which requires HMMR to interact with SACK1D and deliver it to the mitotic spindle, must rapidly facilitate the SACK1 domain of SACK1D to recruit CK1α with such high affinity that both SACK1D and HMMR undergo post-mitotic degradation to release CK1α for other functions. CK1α has been shown to exist in complex with one of the other seven SACK1(A-H) proteins through their SACK1 domains^2^ or GAPVD1^34^^,^^35^ under asynchronous conditions. Where the pool of CK1α that binds SACK1D at the mitotic spindle comes from remains to be determined but this is likely to be mediated by differences in affinity of the SACK1 domains of FAM83 proteins for CK1α. While this study answers many questions about the relationship between SACK1D, HMMR, and CK1α, several questions remain. We show that HMMR binding is essential to the mitosis-specific recruitment of CK1α, however the exact molecular switch which allows CK1α to bind SACK1D at the G2/M transition remains elusive. The localization of CK1α to nascent spindle fibers emanating from centrosomes, combined with the reported involvement of dynein light chain 1 (DYNLL1) demonstrated by other studies, suggests that activation of the CK1α-binding interface on SACK1D may involve a complex regulatory process.^6^ Alternatively, post-translational modification of HMMR or DYNLL1 could be sufficient to facilitate this switch. We have previously shown that the protein kinase activity of CK1α at the mitotic spindle is critical for driving proper spindle orientation and timely resolution of mitosis.^1^ Comprehensive assessment of the substrates modified by mitotic spindle localized CK1α remains to be elucidated. How these substrates play into the observed spindle alignment defects will further consolidate CK1α′s role as a *bona fide* mitotic kinase.

### Limitations of the study

While we highlight several limitations of the study in the “discussion” section above, it should be noted that the residues that we mutated from the HMMR-SACK1D interaction interface are based on structural prediction by Alphafold3. Introducing point mutations on any protein could produce unforeseen structural effects that are difficult to predict or experimentally verify. Therefore, our interpretations derived from point mutations utilized in this study, especially the 9SA mutant (where 9 serine residues on SACK1D were mutated to A to abolish the mitotic phospho-shift), should be considered with caution. Identification of phospho-sites within a cluster of phospho-residues is a technical challenge of mass spectrometry that currently has no easy solutions—therefore, the precise mitotic phosphorylation sites on SACK1D remain to be mapped. It was not possible in this study to identify the precise phospho-degrons on SACK1D or the E3 ligase(s) that mediate the post-mitotic degradation. Additionally, due potentially to its critical importance in many cellular functions, CK1α proved to be KO lethal in many lines tested, including U2OS. As such, comparative knockout studies in U2OS were not possible. DLD-1 was the only cell line where we managed to obtain a CK1α-KO clone that survived and thus we have made use of this clone in this study.

## Resource availability

### Lead contact

Further information and requests for resources and reagents should be directed to and will be fulfilled by the lead contact, Gopal P. Sapkota (g.sapkota@dundee.ac.uk).

### Materials availability

Plasmids generated in this study are available to request from the MRC-PPU reagents webpage (http://mrcppureagents.dundee.ac.uk). U2OS HMMR^−/−^ cells are available from the lead contact with a completed materials transfer agreement.

### Data and code availability

• Original western blot images have been deposited at Mendeley at [Mendeley Data: https://doi.org/10.17632/tgt3vbw2ss.1] and are publicly available. Any other raw data that supports the findings of this study are available from the corresponding author upon reasonable request.
• All raw mass spectrometry data acquired for this study are deposited in the PRIDE database with the accession number PXD013808 [MS Data: https://www.ebi.ac.uk/pride/archive/projects/PXD013808].
• This paper does not report original code.
• Any additional information required to reanalyze the data reported in this paper is available from the lead contact upon request.

## STAR★Methods

### Key resources table

**Table:** 

REAGENT or RESOURCE	SOURCE	IDENTIFIER
**Antibodies**		

Rabbit GFP	MBL	MBL International Cat# 598S, RRID:AB_591816
Sheep GFP	DSTT (University of Dundee)	S268B (Bleed 4)
Rabbit mCherry	Abcam	Abcam Cat# ab213511, RRID:AB_2814891
Rat α-Tubulin	Invitrogen	Thermo Fisher Scientific Cat# MA1-80189, RRID:AB_2210200
Rabbit Vinculin	CST	Cell Signaling Technology Cat# 13901, RRID:AB_2728768
Mouse HMMR	Santa Cruz	Sc-515221
Sheep SACK1D	DSTT (University of Dundee)	SA102
Sheep Ck1α	DSTT (University of Dundee)	SA527 Bleed 3
Sheep Ck1α	DSTT (University of Dundee)	SA527 Bleed 8
Rabbit H3 S10ph	CST	Cell Signaling Technology Cat# 9701, RRID:AB_331535
Rabbit Pericentrin	Invitrogen	Thermo Fisher Scientific Cat# PA5-54109, RRID:AB_2645392
Rabbit Cyclin B1	CST	12231S
Rabbit GAPDH (HRP conjugated)	ProteinTech	Proteintech Cat# HRP-60004, RRID:AB_2737588
Goat anti-Rabbit IgG (H+L) Cross-Adsorbed Secondary Antibody, Alexa Fluor™ 594	Invitrogen	Thermo Fisher Scientific Cat# A-11012, RRID:AB_2534079
Donkey anti-Rat IgG (H+L) Highly Cross-Adsorbed Secondary Antibody, Alexa Fluor™ 647	Invitrogen	(Thermo Fisher Scientific Cat# A78947, RRID:AB_2910635)
Donkey anti-Sheep IgG (H+L) Cross-Adsorbed Secondary Antibody, Alexa Fluor™ 488	Invitrogen	Thermo Fisher Scientific Cat# A-11015, RRID:AB_2534082
Donkey anti-Sheep IgG (H+L) Cross-Adsorbed Secondary Antibody, Alexa Fluor™ 594	Invitrogen	Thermo Fisher Scientific Cat# A-11016, RRID:AB_2534083
Donkey anti-Mouse IgG (H+L) Highly Cross-Adsorbed Secondary Antibody, Alexa Fluor™ 488	Invitrogen	Thermo Fisher Scientific Cat# A-21202, RRID:AB_141607
Goat anti-Mouse IgG (H+L) Cross-Adsorbed Secondary Antibody, Alexa Fluor™ 594	Invitrogen	Thermo Fisher Scientific Cat# A-11005, RRID:AB_2534073
Goat anti-Mouse IgG (H+L) Highly Cross-Adsorbed Secondary Antibody, Alexa Fluor™ Plus 647	Invitrogen	Thermo Fisher Scientific Cat# A32728TR, RRID:AB_2866490

**Chemicals, peptides, and recombinant proteins**		

PhosStop phosphatase inhibitor cocktail tablets	Roche	Cat#4906837001
Benzonase	Merck	Cat#E1014
S-trityl-L-cysteine	Sigma-Aldrich	164739
Polybrene (Hexadimethrine bromide)	Sigma-Aldrich	Cat#107689
Dithiothreitol	Sigma-Aldrich	Cat#D0632

**Critical commercial assays**		

BCA Gold assay	ThermoFisher	Cat#A55860

**Deposited data**		

Phosphoproteomics data	This paper	PRIDE Project ID: PXD013808
Western Blot Data obtained in this study	This paper	Mendeley DOI: DOI: 10.17632/tgt3vbw2ss.1

**Experimental models: Cell lines**		

U2OS	ATCC	Cat# HTB-96
DLD-1	ATCC	Cat# CCL-221

**Oligonucleotides**		

esiRNA HMMR	Merck	Cat#EHU060291
esiRNA FAM83D/SACK1D	Merck	Cat#EHU084291
universal negative control 1	Merck	Cat#SIC001

**Recombinant DNA**		

pCMV5-gag-pol	Cell Biolabs	Cat# RV-111
pCMV5-VSV-G	Cell Biolabs	Cat# RV-110
pBabeD HMMR-eGFP	MRC PPU Reagents & Services	DU79082
pBABED HMMR^ΔK76-E91^-mCherry [Δexon4]	MRC PPU Reagents & Services	DU79095
pBABED HMMR (L374A F375A)-eGFP	MRC PPU Reagents & Services	DU79287
pBabeD GFP-SACK1D	MRC PPU Reagents & Services	DU28538
pBabeD GFP SACK1D aa1-514	MRC PPU Reagents & Services	DU75603
pBabeD mCherry-CK1α N141A	MRC PPU Reagents & Services	DU69774
pBabeD mCherry-CK1α	MRC PPU Reagents & Services	DU69773
pBabeD GFP-SACK1D aa514-585	MRC PPU Reagents & Services	DU75604
pBabeD GFP-SACK1D 9SA [S472A, S473A, S474A, S477A, S478A, S479A, S480A, S482A, S483A]	MRC PPU Reagents & Services	DU69765
pBabeD GFP SACK1D x2ML [M545A, L546A, M548A, L549A] DU75609	MRC PPU Reagents & Services	DU75609
pBabeD P U6 HMMR Nter KI Sense A	MRC PPU Reagents & Services	DU60921
pX335 HMMR Nter KI Antisense A	MRC PPU Reagents & Services	DU60934

**Software and algorithms**		

FIJI	ImageJ	https://imagej.net/
GraphPad Prism v10.6.1	GraphPad	https://www.graphpad.com/scientific-software/prism/
Alphafold3	Google	https://alphafoldserver.com
Chimera X	UCSC	https://www.cgl.ucsf.edu/chimera/

**Other**		

Leica Stellaris 8 Falcon FLIM confocal microscope	Leica	https://www.leica-microsystems.com/products/confocal-microscopes/p/stellaris-8-falcon/
DeltaVison Elite widefield microscope	GE	https://www.imsol.co.uk/deltavision-elite/
Pierce™ ECL Western Blotting Substrate	Thermo Scientific	Cat#32106
Lipofectamine RNAiMax	Thermo Fisher	Cat#13778075
Triton™ X-100	Merck	Cat#X100
ProLong® Glass anti-fade reagent with NucBlue	Life Technologies	Cat#P36981
Amersham™ Protran 0.45 NC nitrocellulose Western blotting membrane	Cytiva	Cat#10600002

### Experimental model and study participant details

#### Cell lines

Human osteosarcoma U2OS (female) and colorectal adenocarcinoma DLD-1 (male) cells were grown in Dulbecco’s modified Eagle’s medium (DMEM; Gibco) containing 10% (v/v) Foetal Bovine Serum (FBS; Hyclone), penicillin (100 U/ml; Lonza), streptomycin (0.1 mg/ml; Lonza), l-glutamine (2 mM; Lonza) and Plasmocin (5ug/ml, Invivogen) and cultured at 37°C, 5% CO2 in a humidified tissue culture incubator. All cells used in this study were verified to be free from mycoplasma contamination by PCR and/or Immunofluorescence. Cells were exposed to different stimuli and compounds as described in the appropriate figure legends prior to lysis. For retroviral-based infections, cells were transduced with retroviruses as described previously.^36^^,^^37^

### Method details

#### Retroviral constructs

Recombinant DNA procedures were performed using standard protocols as described previously.^2^^,^^38^ Human wild-type (WT) SACK1D, HMMR and CSNK1A1 or appropriate mutants were sub-cloned into pBabeD vectors (pBabeD denotes a Dundee-modified version of the pBABE Puro vector). SACK1D and HMMR constructs harbour a Green Fluorescence Protein (GFP) tag at the N- and C-terminus, respectively, unless indicated otherwise. All constructs are available to request from the MRC-PPU reagents webpage (http://mrcppureagents.dundee.ac.uk), and the unique identifier (DU) numbers indicated below provide direct links to sequence information and the cloning strategy used. The following constructs were generated: pBabeD HMMR-eGFP (DU79082), pBABED HMMR^ΔK76-E91^-mCherry [Δexon4] (DU79095), pBABED HMMR (L374A F375A)-eGFP (DU79287), pBabeD GFP-SACK1D (DU28538), pBabeD GFP-SACK1D 9SA [S472A, S473A, S474A, S477A, S478A, S479A, S480A, S482A, S483A] (DU69765), pBabeD GFP SACK1D aa1-514 (DU75603), pBabeD GFP SACK1D x2ML [M545A, L546A, M548A, L549A] (DU75609), pBabeD GFP-SACK1D aa514-585 (DU75604) pBabeD mCherry-CK1α (DU69773) and pBabeD mCherry-CK1α N141A (DU69774). Constructs were sequence-verified by the DNA Sequencing Service, University of Dundee (http://www.dnaseq.co.uk). For amplification of plasmids, 1 μl of the plasmid was transformed into 30 μL of *Escherichia coli* DH5α competent bacteria (Invitrogen) on ice, incubated at 42°C for 45 s and then returned to ice for 2 min, before plating on LB-agar medium plate containing 100 μg/ml ampicillin. LB plates were inverted and incubated for 16 h at 37°C. Following incubation, a single colony was picked and used to inoculate 4 ml of LB medium containing 100 μg/ml ampicillin. Cultures were grown for 18 h at 37°C in a bacterial shaker (Infors HT). Plasmid DNA was purified using a Qiagen mini-prep kit as per the manufacturer’s instructions. The isolated DNA yield was subsequently analysed and quantified using a Nanodrop 1,000 spectrophotometer (Thermo Scientific).

#### Generation of U2OS HMMR^-/-^ cells

To generate *HMMR*^−*/*−^ knockout by CRISPR/Cas9 genome editing, U2OS cells were transfected with vectors encoding a pair of guide RNAs (pBabeD P U6 HMMR Nter KI Sense A and pX335 HMMR Nter KI Antisense A) targeting around the first exon of *HMMR* (1 μg each) (DU60921 and DU60934). 16 h post-transfection, cells were selected in puromycin (2 μg/ml) for 48 h. The transfection process was repeated one more time. For the acquisition of single-cell clones of knockouts, single cells were isolated by fluorescence-activated cell sorting (FACS) using an Influx cell sorter (Becton Dickinson). Single cell clones were plated on individual wells of two 96-well plates, pre-coated with 1% (w/v) gelatin to help cell adherence. Viable clones were expanded, and successful knockout at the target locus was confirmed by both Western blotting and genomic DNA sequencing.

#### Cell lysis and immunoprecipitation assays

Media was gently aspirated, and cells lysed directly in lysis buffer (50 mM Tris–HCl pH 7.5, 150 mM NaCl, 1 mM EDTA and 1% Triton-X), supplemented with 1x cOmplete™ protease inhibitor cocktail (Roche), PhosSTOP complete phosphatase inhibitor (Roche) and Benzonase (E1014, Merck). Cell extracts were either clarified and processed immediately, or snap-frozen in liquid nitrogen and stored at −20°C. Protein concentrations were determined in a 96-well format using the BCA Gold assay (ThermoFisher).

For Immunoprecipitations (IPs), clarified extracts were normalised in lysis buffer to typically 0.75-1 mg/ml protein. After input aliquots were collected, lysates were incubated O/N at 4°C with protein G-sepharose beads coupled to the antibody of interest, on a rotating wheel. For anti-GFP IPs, GFP nanobody conjugated to Sepharose and for anti-CK1α IPs, anti-CK1α antibody-coupled sepharose beads were used, using in-house generated anti-CK1α antibody. Following incubation, beads were pelleted and were washed once in lysis buffer and 2–3 times in PBS. For elution, beads were resuspended in 1× SDS sample buffer (BioRad) and incubated at 95°C for 5 min.

#### Cell synchronization and washout assays

For synchronisation, cells were arrested at prometaphase with kinesin Eg5 inhibitor S-trityl-L-cysteine (STLC; 5 μM) for 16 h, before rounded/floating mitotic cells were isolated through mitotic shake-off. Collected mitotic cells were centrifuged and media aspirated before lysis in lysis buffer. Phospho-S10 Histone H3 (H3 S10ph) immunoblotting was employed as a marker of efficient mitotic arrest. For washout assays, cells that were synchronized with STLC were centrifuged and washed once in PBS before being plated in complete culture medium. Cells were then collected at indicated time points by media centrifugation at early time points where cells were still in suspension or direct lysis on plates where cells had attached.

#### SDS-PAGE and western blot

Reduced protein extracts (typically 10–20 μg protein, Lamelli with DTT) or IPs were resolved on 4–12% NuPAGE bis-tris precast gradient gels (Invitrogen) by electrophoresis. Separated proteins were subsequently transferred onto nitrocellulose membranes (Amersham), before membranes were blocked in 5% (w/v) non-fat milk powder (Marvel) in TBS-T (50 mM Tris–HCl pH 7.5, 150 mM NaCl, 0.2% (v/v) Tween-20) and incubated overnight at 4°C in 5% milk TBS-T with the appropriate primary antibody. Membranes were then washed for 3 × 10 min with TBS-T before incubating with HRP-conjugated secondary antibodies in 5% milk TBS-T for 1 h at RT. Membranes were then washed for 3 × 10 min with TBS-T before detection with enhanced chemiluminescence reagent (Invitrogen) and imaged using the ChemiDoc™ system (Bio-Rad).

#### siRNA transfection

U2OS cells were transfected with esiRNAs (600ng in 2ml in a well of a 6 well plate) targeting HMMR (EHU060291, Merck), FAM83D/SACK1D (EHU084291, Merck) or the universal negative control 1 (SIC001, Merck) using Lipofectamine RNAiMax Reagent (13778075, Thermo Fisher). Lipid–siRNA mixtures were incubated in Opti-MEM (Gibco) for 5 min before being added dropwise to cells. Cells were collected for analysis after 24 h with co-treatment with STLC (5 μM) or DMSO added for 16 h before lysis as required.

#### Confocal and widefield immunofluorescence microscopy

Cells were seeded onto glass coverslips and treated/transfected as described above or in figure legends. Media was gently aspirated before fixing in 4% (w/v) paraformaldehyde (PFA) in 1 PHEM buffer (60 mM PIPES, 25 mM HEPES, 10 mM EGTA, and 4 mM MgSO4·7H20) for 10 min at 37°C. Cells were washed twice in PBS, followed by incubation in PBS at 4°C or stained immediately. Cells were permeabilised for 5 min in 0.1% Triton in PBS. Following permeabilization, cells were blocked in PBS containing 5% (w/v) BSA for 1 h. Where appropriate, coverslips were then incubated with primary antibody in PBS/BSA 0.1% Triton (at dilutions indicated in the antibody table) at 37°C for 1 h. Cells were washed for a minimum of 3 × 10 min in PBS/BSA before incubation with the secondary AlexaFluor-conjugated antibody in PBS/BSA 0.1% Triton (1:1000) for 60 min at RT protected from light. Coverslips were subsequently washed for 1 × 10 min each in PBS 0.1% Triton, PBS and MilliQ Water, then dried and mounted on glass microscopy slides using ProLong® Glass anti-fade reagent with NucBlue (Life Technologies). Coverslips were left to set overnight at 4°C before analysis on a Leica Stellaris 8 Falcon FLIM confocal microscope using an HC PL APO 63x/1.40 OIL CS2 lens. Alternatively, slides were imaged on a DeltaVison Elite widefield microscope using x100 PlanApoN 1.42 NA objective at 1 × 1 binning and a CoolSNAP HQ camera (Photometrics).

#### Immunofluorescence microscopy analysis

All image analysis was performed in FIJI. For quantification of CK1α, SACK1D and HMMR on the mitotic spindle for siRNA studies (Figure 1D), the mean pixel intensities of proteins of interest were calculated by designating a circular region of interest (ROI) around the DNA of the cell and subtracting the mean pixel intensity of the background (ROI) outside the first region but inside the cell. For quantification of HMMR and CK1α in HMMR rescue experiments (Figure 2A) and GFP-SACK1D^514-585^ rescue experiments (Figure 4A) mean pixel in intensity was obtained for an ROI determined by α-tubulin staining (MaxEntropy Threshold) and subtracting the mean pixel intensity of the background (ROI) outside the first region but inside the cell. For colocalization assays (Figure 3C), Manders overlap coefficients were obtained with the JACoP plugin for proteins as indicated. Macros used are supplied in supplemental information.

#### Mitotic spindle alignment assay

To determine mitotic spindle orientation, cells were stained with pericentrin antibody to indicate centrosomes and the position of each centrosome relative to the other recorded in prometaphase-metaphase cells. A minimum of 24 cells per condition were recorded spread over three independent experiments.

#### Antibodies

The antibodies used in this study are listed in below tables. The source, catalog number and dilution factor used for each antibody are provided. The in-house antibodies were generated by the MRC PPU Reagents and Services, University of Dundee as described previously.^2^ For HRP-coupled secondary antibodies, goat anti-rabbit-IgG (cat.: 7074, 1:1000) was from CST, and rabbit anti-sheep-IgG (cat.: 31480, 1:1,000) and goat anti-mouse-IgG (cat.: 31430, 1:1,000) were from Thermo Fisher Scientific.

**Table:** Primary antibodies used in this study

Antigen	Source	Cat. Number	Dilution	Figure	Species
GFP	MBL	589	1:1000 (WB)	4B	Rabbit
GFP	DSTT (University of Dundee)	S268B (Bleed 4)	1:1000 (WB) 1:500 (IF)	1A, 3B, 5D&E	Sheep
mCherry	Abcam	ab213511	1:1000 (WB) 1:500 (IF)	1A, 2A & 5A	Rabbit
α-Tubulin	Invitrogen	MA1-80189	1:1000 (WB)	1A&B, 2B&D &3C	Rat
Vinculin	CST	13901S	1:5000 (WB)	1C, 2C, 3E, 4C&E	Rabbit
HMMR	Santa Cruz	Sc-515221	1:1000(WB) 1:500 (IF)	1B&C, 2A, B&C, 3B&C, 4B, C, D &E,	Mouse
SACK1D	DSTT (University of Dundee)	SA102	1:1000 (WB) 1:500 (IF)	1C, 2C, 3E, 4B, C, D&E, 5A, &C & S3B	Sheep
Ck1α	DSTT (University of Dundee)	SA527 Bleed 3	1:1000 (WB) 1ug/ml (IP)	1C, 2C, 3B&E, 4C&D & 5A	Sheep
Ck1α	DSTT (University of Dundee)	SA527 Bleed 8	1:500 (IF)	2B, & 3C	Sheep
H3 S10ph	CST	9701S	1:1000 (WB)	1C, 2C, 3E, 4B, C, D&E & 5C, D&E	Rabbit
Pericentrin	Invitrogen	PA5-54109	1:50 (IF)	1B & 2D	Rabbit
Cyclin B1	CST	12231S	1:1000 (WB)	5A	Rabbit
GAPDH (HRP conjugated)	ProteinTech	HRP-60004	1:5000 (WB)	3B&D, 4D, 5A,C,D&E, & EV3	Rabbit

**Table:** Secondary antibodies used for immunofluorescence in this study

Target Species	Raised Species	Wavelength	Source	Catalog Number	Dilution Factor
Rabbit	Goat	594nM	Invitrogen	A11012	1:1000
Rat	Donkey	647nM	Invitrogen	A78947	1:1000
Sheep	Donkey	488nM	Invitrogen	A11015	1:1000
Sheep	Donkey	594nM	Invitrogen	A11016	1:1000
Mouse	Donkey	488nM	Invitrogen	A21202	1:1000
Mouse	Goat	594nM	Invitrogen	A11005	1:1000
Mouse	Goat	647nM	Invitrogen	A32728	1:1000

#### Alphafold3 analysis

The canonical sequences of human HMMR and FAM83D/SACK1D proteins obtained from UNIPROT were uploaded to the Alphafold3 webserver at https://alphafoldserver.com/. The highest confidence prediction was then cross-referenced with other predictions provided using Chimera X (UCSC) and predicted interactions determined by PAE analysis of bonds forming between predicted intermolecular contacts.

### Quantification and statistical analysis

Statistical analysis was carried out in GraphPad (Prism) and was used to generate plots and analyse data by unpaired Student’s t-test or one-way ANOVA as appropriate following normality tests. A P-value of < 0.05 was deemed significant. Unless indicated otherwise, all experiments are representative of at least three independent biological replicates. Where appropriate, the data from all three replicates is deposited in at Mendeley Data linked to this manuscript.

All details about the sample size n, biological replicates, data shown (mean and S.D.) and p-values are given in the main results text, Figures, Figure legends, and the Supplemental Figures and their legends.

## Acknowledgments

GPS is supported by the UKRI Medical Research Council (grants MC_UU_00018/6 and MC_UU_00038/6) and Boehringer Ingelheim through the Division of Signal Transduction Therapy. We thank the Sapkota lab members for critical appraisal of the data. We thank E. Allen, A. Muir, S. Dalglish, E. Webster, and J. Stark for help and assistance with tissue culture, the staff at the DNA Sequencing services (School of Life Sciences, University of Dundee), and the cloning and antibody teams within the MRC-PPU Reagents and Services (University of Dundee, coordinated by J. Hastie). We thank the staff at the flow cytometry facility (School of Life Sciences, University of Dundee) for their invaluable support. We thank the MRC PPU mass spectrometry service team for their help with the project. We thank members of the Dundee Imaging Facility (University of Dundee, coordinated by P. Appleton) for microscopy support.

## Author contributions

T.N.C. performed most of the experiments, collected and analyzed data, and contributed to the writing of the manuscript; N.K.N., L.J.F., K.D., and S.B. performed some experiments and collected and analyzed data; N.T.W. performed cloning; T.J.M. designed the strategies for and generated the CRISPR/Cas9 constructs used in this study; G.P.S. conceived the project, analyzed data, and contributed to the writing of the manuscript.

## Declaration of interests

The Sapkota laboratory receives or has received sponsored research support from Amgen Inc., Boehringer Ingelheim, GlaxoSmithKline, and Johnson & Johnson. The authors have no other conflicts of interest to declare.
