# Supplementary material for: HMMR/RHAMM recruits SACK1D/FAM83D-CK1α complex at the mitotic spindle to control spindle alignment
Source: iScience. 2025 Dec 12;29(1):114417. doi: 10.1016/j.isci.2025.114417 (PMC12803952; doi:10.1016/j.isci.2025.114417)
Supplement: Document S1. Figures S1–S3 [file mmc1.pdf]

iScience, Volume *29*

Supplemental information

HMMR/RHAMM recruits

SACK1D/FAM83D-CK1α complex at

the mitotic spindle to control spindle alignment

Tyrell N. Cartwright, Naveen K. Nakarakanti, Karen Dunbar, Luke J. Fulcher, Selina Bader, Nicola T. Wood, Thomas J. Macartney, and Gopal P. Sapkota

Supplementary Figures and Legends

Figure S1: Confirmation of HMMR knockout and SACK1D localization in HMMR$^{-/-}$ cells.

Figure S2: Alphafold3 prediction of SACK1D-HMMR complex depicting the binding interface

Figure S3: Identification of the phospho-sites on SACK1D responsible for the mitotic electrophoretic mobility shift.

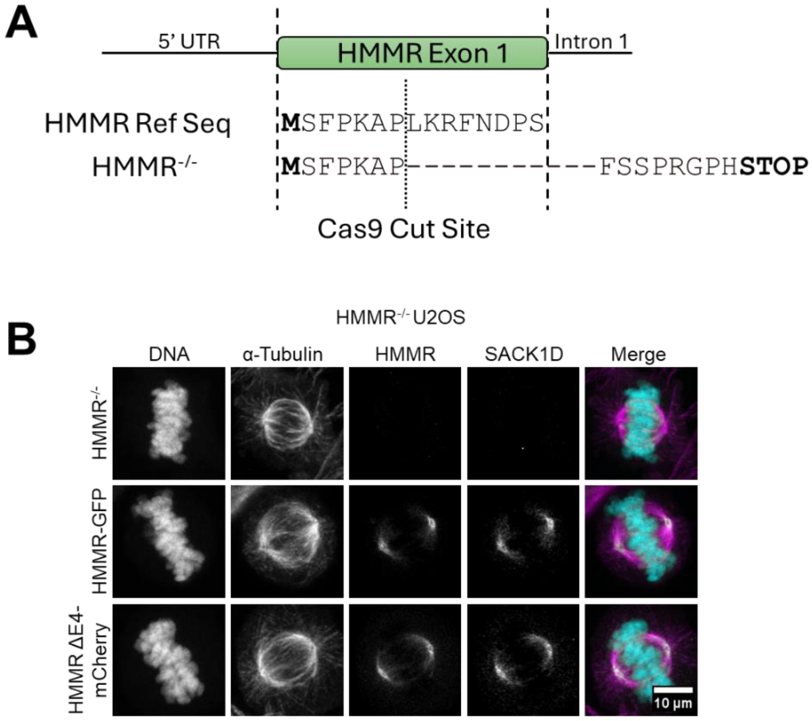

Figure S1: **Confirmation of HMMR knockout and SACK1D localization in HMMR$^{-/-}$ cells.**

A. Schematic of the sequencing results confirming a deletion in exon 1 of HMMR.

B. Representative confocal IF microscopy images of HMMR$^{-/-}$ U2OS cells either left untransduced or transduced with retroviruses to stably express HMMR-GFP or HMMR-GFP lacking exon 4 (ΔE4-GFP) and stained for HMMR, SACK1D and α-tubulin as indicated.

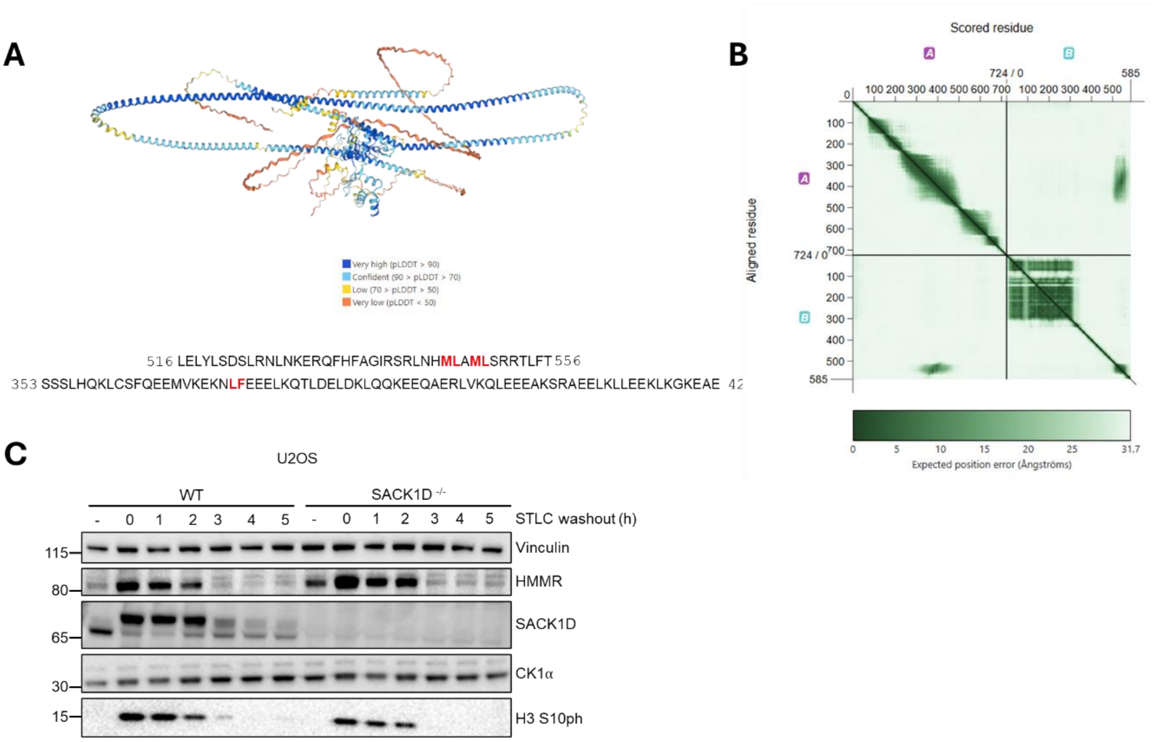

Figure S2. Alphafold3 prediction of SACK1D-HMMR complex depicting the binding interface

A. 3D representation of the predicted structure of SACK1D bound to HMMR. Colours indicate predicted Local Distance Difference Test values according to the legend provided. Amino acid sequences from SACK1D (top) and HMMR (bottom) at the interface, with the residues predicted to make molecular contacts highlighted in red.

B. Predicted aligned error (PAE) plot of SACK1D against HMMR.

C. Representative western blot analysis with the indicated antibodies of lysate inputs from an STLC washout assay performed in WT U2OS and SACK1D$^{-/-}$ U2OS cells and lysed at indicated timepoints (h) following mitotic arrest.

A Phospho-peptides in SACK1D$^{GFP/GFP}$ KI U2OS cells Nocodazole vs. AS controls

Phospho-peptides Identified in Mitotic but not AS Extracts	Human SACK1D Phosphorylation Residue	Conserved in Mice?	Conserved in Xenopus?
LARLsSTPR	Ser338	YES	YES
LARLSStPR	Thr340	YES	YES
GTQStEGSPVSKmSVSR	Thr459	NO	NO
YLGtPHLELYLSDSLR	Thr513	YES	NO

B Phospho-peptides in SACK1D$^{GFP/GFP}$ KI U2OS cells STLC vs. AS controls

Phospho-peptides Identified in Mitotic but not AS Extracts	HumanSACK1D Phosphorylation Residue	Conserved in Mice?	Conserved in Xenopus?
RLsSTPRK	Ser338	YES	YES
RLSStPRK	Thr340	YES	YES
GTQStEGSPVSK	Thr459	NO	NO

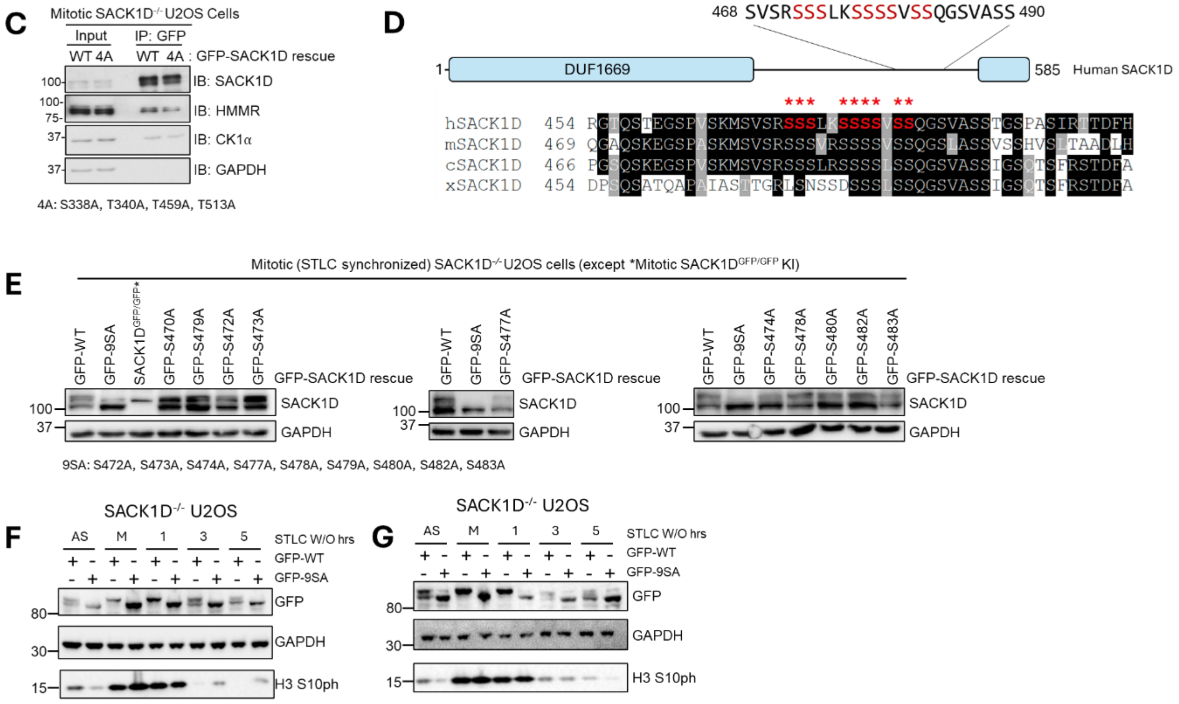

Figure S3. Identification of the phospho-sites on SACK1D responsible for the mitotic electrophoretic mobility shift.

A. Table showing the phospho-peptides on SACK1D identified by mass-spectrometry in anti-GFP IPs from nocodazole-synchronized mitotic SACK1D$^{GFP/GFP}$ knockin U2OS cell extracts that were absent in asynchronous (AS) extracts. The residue numbers corresponding to human SACK1D and whether they are conserved in mouse and *Xenopus* are indicated.

B. As in (A), except the cells were synchronised in mitosis by treating them with STLC.

C. The 4 phospho-residues identified in (A) were mutated to Ala (4A) and restored in SACK1D$^{-/-}$ U2OS cells as indicated. STLC-synchronized mitotic extracts or anti-GFP IPs were subjected to western blot analysis with the indicated antibodies. The GFP-SACK1D-4A mutant did not alter the mitotic electrophoretic mobility shift of SACK1D compared to GFP-SACK1D-WT suggesting there are more mitotic phospho-sites on SACK1D that were not identified by mass-spectrometry.

D. Schematic showing the location of the conserved serine-rich cluster region on SACK1D that we considered as a potential region that could be hyperphosphorylated in mitosis to account for

the electrophoretic mobility shift. The indicated 9 Ser residues were chosen to mutate to Ala either individually or collectively. Sequence alignment of this region with human, mouse, chicken and Xenopus SACK1D was performed by Clustal Omega and image generated using BoxShade Server.

E. Western blot analysis of single Ser to Ala point mutations and all 9 Ser to Ala (9SA) GFP-SACK1D mutants showing that only 9SA mutations result in the complete collapse of the mitotic SACK1D band shift. All mutants were retrovirally transduced in SACK1D$^{-/-}$ U2OS cells. * Indicates U2OS SACK1D$^{GFP/GFP}$ knockin mitotic extract included as a positive control for the observed electrophoretic mobility shift.

F-G. Replicates of western blot analysis in Figure 5D with the indicated antibodies of lysate inputs from an STLC washout assay using WT or SACK1D$^{-/-}$ U2OS cells expressing the indicated GFP-SACK1D constructs in asynchronous and mitotic cells (5 μM STLC for 16 h).
